# Post-transcriptional Regulation of Colorectal Cancer: A Focus on RNA-Binding Proteins

**DOI:** 10.3389/fmolb.2019.00065

**Published:** 2019-08-07

**Authors:** Jennyfer M. García-Cárdenas, Santiago Guerrero, Andrés López-Cortés, Isaac Armendáriz-Castillo, Patricia Guevara-Ramírez, Andy Pérez-Villa, Verónica Yumiceba, Ana Karina Zambrano, Paola E. Leone, César Paz-y-Miño

**Affiliations:** Facultad de Ciencias de la Salud Eugenio Espejo, Centro de Investigación Genética y Genómica, Universidad UTE, Quito, Ecuador

**Keywords:** colorectal cancer, RBPs, post-transcriptional regulation, oncogene, tumor suppressor

## Abstract

Colorectal cancer (CRC) is a major health problem with an estimated 1. 8 million new cases worldwide. To date, most CRC studies have focused on DNA-related aberrations, leaving post-transcriptional processes under-studied. However, post-transcriptional alterations have been shown to play a significant part in the maintenance of cancer features. RNA binding proteins (RBPs) are uprising as critical regulators of every cancer hallmark, yet little is known regarding the underlying mechanisms and key downstream oncogenic targets. Currently, more than a thousand RBPs have been discovered in humans and only a few have been implicated in the carcinogenic process and even much less in CRC. Identification of cancer-related RBPs is of great interest to better understand CRC biology and potentially unveil new targets for cancer therapy and prognostic biomarkers. In this work, we reviewed all RBPs which have a role in CRC, including their control by microRNAs, xenograft studies and their clinical implications.

## Introduction

Worldwide, every year an estimated 1.8 million new cases of colorectal cancer (CRC) are diagnosed, setting it in the third place of the most common malignant tumor, and consequently a major health care problem (Gao et al., 2015; Bray et al., 2018). Heretofore, most studies in CRC biology have been focused on DNA-related aberrations (e.g., mutation, methylation changes, DNA copy number alterations, loss of genomic stability, etc.), leaving the post-transcriptional processes under-studied. However, post-transcriptional alterations play a significant role in the preservation of tumor cells by modulating every hallmark in cancer (Lukong et al., 2008; Morris et al., 2012; Paz-Y-Mino et al., 2015; Wurth et al., 2016; Martinez-Useros et al., 2017).

RNA biology represents an under-investigated aspect of cancer; this is puzzling considering that pleiotropic changes in the transcriptome are a key feature of cancer cells (Wurth and Gebauer, 2015). RNA binding proteins (RBPs) are relevant because they are part of post-transcriptional RNA regulons. These RNA regulons are formed by Ribonucleoproteins (RNP) which interact with other trans-elements, non-coding RNAs, metabolites and untranslated sequence elements found within the mRNAs (USER). These RNP complexes control the expression of hundreds to thousands mRNAs of functionally related proteins from the transcription to translation process, allowing the cell to respond to several stimuli with such a great agility ensuring cellular homeostasis (Keene, 2007; Wurth, 2012; Iadevaia and Gerber, 2015; Wurth and Gebauer, 2015). The complex interaction of RBPs and their RNA partners (e.g., mRNAs or miRNAs) are achieved through RNA-recognition domains which increases the specificity and affinity of these interactions. While there is much progress still to understand such interactions, some of these domains have been ully characterized: the RNA-recognition motif (RRM), the zinc finger motif, and the K-homology domain (Iadevaia and Gerber, 2015). RBPs are able to control every aspect of RNA metabolism: capping, splicing, polyadenylation, nucleocytoplasmic transport, stability, translation, and degradation of mRNA (Burd and Dreyfuss, 1994; Lukong et al., 2008; Kechavarzi and Janga, 2014). As a result, when any RBP is altered this affect either its mRNA affinity or its subcellular localization, disturbing cellular homeostasis (Iadevaia and Gerber, 2015). In this regard, RBPs are emerging as critical modulators of every hallmark of cancer, and still very little is known about their cancer related molecular functions and targets (Wurth and Gebauer, 2015; Hentze et al., 2018).

Hentze et al. compiled all published RNA interactomes into RBP supersets, they stringently curated and updated the annotations of RBPs identified from several sources. Finally, a list of 1,393 RBPs was retrieved in humans and only a few have been implicated in the carcinogenic process and even much less in CRC (Hentze et al., 2018). The identification of RBPs will provide a better understanding of tumor biology and potentially unveil new targets for cancer therapy and prognostic biomarkers. In this work, we reviewed all RBPs having a role in CRC, including their control by microRNAs (miRNAs), xenograft studies and their clinical implications.

## LIN28

### General Features

LIN28 is an evolutionarily conserved RBP and an emerging oncogenic driver (Zhang et al., 2018). Mammals produce two LIN28 paralogs, *LIN28A* and *LIN28B* which are separately or jointly involved in various biological functions; including metabolism development, tissue regeneration, and oncogenesis (Tu et al., 2015; Wang T. et al., 2015; Jiang and Baltimore, 2016; Wang et al., 2016; Pereira et al., 2017). Human *Lin28A* is located on chromosome 1p36 and encodes a protein of 209 amino acids whereas *Lin28B* is on chromosome 6q16.3 and its protein is composed of 250 amino acids. In addition, LIN28A is predominantly localized in the cytoplasm, whereas LIN28B resides exclusively in the nucleus. Interestingly, both proteins are expressed mainly in the cytoplasm in CRC (Guo et al., 2006; Wang et al., 2016).

LIN28 proteins have two cold shock domains and retroviral-type Cys-Cys-His-Cys (CCHC) zinc fingers that confer RNA-binding ability. These proteins also modulate the let-7 family of miRNAs, which consists of 12 members frequently deleted in human cancers and considered as tumor suppressors (Zhou et al., 2013; Triboulet et al., 2015; Wang S. et al., 2015; Jiang and Baltimore, 2016; Jiang et al., 2017). Both proteins inhibit biogenesis and induce the degradation of the let-7 family. LIN28B interacts with pri-let-7 (primary-miRNAs) and inhibits its processing by the Microprocessor complex, whereas LIN28A blocks the pre-let-7 (the hairpin structure formed by the cleavage of DROSHA/DGCR8 enzymes) processing by DICER1 via TUT4 recruitment (Kim et al., 2014; Wang et al., 2016). Conversely, there is a double-negative feedback loop, where the 3′UTR of LIN28A/B is recognized by let-7 miRNA. Thus, once let-7 miRNA binds to the 3′UTR the expression of these proteins is inhibited (Wang T. et al., 2015). Indicating that when *LIN28A/B* are expressed let-7 is not (Piskounova et al., 2011; Wang T. et al., 2015).

Both LIN28A and LIN28B could enhance colon cancer cells proliferation but mechanically their mode of action is different. *LIN28A* overexpression promotes the transition from S to G2/M phase, whereas constitutive expression of *LIN28B* enables the shift of cell cycle phases (from G1 to S phase and from S to G2/M phase) (Wang et al., 2016). Activation of *LIN28* in different primary tumors leads to translational enhancement or suppression of cancer-related mRNAs (e.g., IGF2 and MYOD1 mRNAs) (Viswanathan and Daley, 2010; Rappaport et al., 2017). Both *LIN28A* and *LIN28B* are expressed in about 30% of colorectal tumors, but the expression level of *LIN28B* is higher compared to *LIN28A* (King et al., 2011a,b; Wang et al., 2016).

### Xenograft Studies

*LIN28B* knockdown in cancer cells reduces their proliferative and invasive abilities *in vitro* and inhibits both primary and metastatic tumor growth *in vivo* (Jiang et al., 2017). LIN28B increases cancer cell invasion in intestinal and colorectal adenocarcinomas in murine models (Jiang et al., 2017). Also*, LIN28* overexpressed tumors exhibited augmented areas of moderate differentiation and increased glandular formation and mucin production; in contrast to wild-type tumors that are poorly differentiated and rarely exhibit mucinous (King et al., 2011a). In addition, Tu et al. experimentally demonstrated that LIN28 cooperates with APC in accelerating neoplastic lesions formation in *Apc*^*Min*/^^+^ mice. APC alterations (or other changes that target WNT signaling) occur in most colon tumors, which could be upregulating the expression of *LIN28B*. This may be mediated by MYC, a transcriptional target of the canonical WNT signaling (Mayr et al., 2007; King et al., 2011a; Zhou et al., 2013; Wang et al., 2016; Pereira et al., 2017). It has also been demonstrated that LIN28/let-7 axis promotes invasive intestinal adenocarcinoma in murine models by interacting with the WNT pathway (Piskounova et al., 2008; Tu et al., 2015; Voutsadakis, 2018).

### miRNAs Control

To date, besides let-7, several miRNAs have been reported to repress LIN28A/LIN28B translation once they bind to their 3′UTR, such as miR-9, miR-26a, miR-27, miR-30, miR-125, miR-181, and miR-212. In cancer cells, these miRNAs are under-expressed due to *LIN28A/B* overexpression (Wang T. et al., 2015).

### Clinical Relevance

LIN28A/LIN28B influence the clinical outcome in patients by enhancing tumor aggressiveness and early metastasis (Mayr et al., 2007; Viswanathan et al., 2009; King et al., 2011a; Wang et al., 2016; Pereira et al., 2017). Irregular *LIN28A/B* expression is usually correlated with poor survival. Several studies have demonstrated that high levels of *LIN28* in colon tumors are associated with advanced tumor stages and increased probability of tumor recurrence (King et al., 2011a; Madison et al., 2013; Jiang and Baltimore, 2016; Zhang et al., 2018). King et al. have found that LIN28B protein levels are increased in CRC patients promoting cancer progression and metastasis (King et al., 2011a). In addition, over-expression of *LIN28A/LIN28B* could enhance chemotherapy sensitivity of HCT116 cells to 5-Fu via different mechanisms (Wang et al., 2016). LIN28 may also serve as a predictive biomarker for chemotherapy in patients with colon cancer (King et al., 2011a; Pang et al., 2014; Wang T. et al., 2015; Wang et al., 2016; Jiang et al., 2017). All RBPs and their CRC-related features are listed in Table 1.

**Table 1:** A summary of all CRC-related RBPs reviewed in this work: miRNAs control and their clinical relevance.

**RBP**	**Control by miRNAs**	**Clinical relevance**
LIN28	miR-let-7, miR-26a, miR-181, miR-9, miR-30, miR-125, miR-212 and miR-27	- Aberrant expression correlates with reduced patient survival. - Predictive biomarker for chemotherapy.
MSI	miR-137	- High expression correlates with increased metastatic risk and poorer survival. - Promotes resistance to 5-FU.
ELAVL1	miR-519 and miR-22	- High expression correlates with malignancy and multidrug resistance.
QKI	miR-574-5p and miR-155	- Low expression correlates with poorer prognosis. - Could predict recurrence and prognosis.
RBM3		- Promotes resistance to chemotherapy
CELF1	miR-503	
IGF2BPs		- Overexpression correlates to unfavorable clinical outcomes: early dissemination, poor response to the therapy, increased tumor aggressiveness, and short survival.
ESRP1		- Overexpression associates with a favorable overall survival outcome.
TTP	miR-29a	- Downregulation correlates with poor prognosis, tumor aggressiveness, and necrosis.
hnRNPs		- Poor prognosis marker.
TIA1	miR-19a	- Increased numbers of TIA-1 positive TILs is associated with an improved clinical outcome. - TIA1 can also be used to supplement prognostic information related to TNM stage and adjuvant therapy.
KHDRBS1		- KHDRBS1 nuclear localization and overexpression is correlated with poor tumor differentiation, advanced T stage, lymph node involvement, and distant metastasis.
CPEB4	miR-203	- Overexpression correlates with tumor progression and poor overall survival.
CSDE1		- Overexpression is associated to poor prognosis.

## MSI

### General Features

In humans, Musashi RNA binding protein (MSI) is composed of two isoforms: MSI1 and MSI2 (Voutsadakis, 2018). *MSI1* and *MSI2* genes are evolutionarily conserved given their 75% amino acid sequence. These two proteins are placed on chromosome 12q24 (*MSI1*) and 17q22 (*MSI2*). They comprise two RNA recognition motif (RRMs) domains which bind to (G/A)U1-3(AGU) motifs in the 3′-UTR of their target mRNAs (Sakakibara et al., 2001; Okano et al., 2005; Glazer et al., 2012). They can be regulated by ELAV1 by maintaining the stabilization of their mRNAs, as well as by tumor suppressor miRNAs (Gao et al., 2015). Musashi emerges as a critical player in controlling multiple targets that form networks from where MSI1 and MSI2 are able to regulate cell death, differentiation, and cell cycle (de Sousa Abreu et al., 2009; Guinney et al., 2015; Kharas and Lengner, 2017). They are also key oncogenic players in promoting intestinal transformation (Wang S. et al., 2015; Kharas and Lengner, 2017). Musashi may also co-operate with LIN28 by binding and inhibiting some mRNAs. Besides, Musashi represses translation of Numb (an inhibitor of the NOTCH pathway), APC, PTEN, and P21, but upregulates WNT pathway at transcriptional level when Numb is inhibited (Qiao and Wong, 2009; Lan et al., 2015; Wang S. et al., 2015; Voutsadakis, 2018).

### Xenograft Studies

Several studies have shown the potential therapeutic target of MSI1 given that when *MSI1* is knockdown tumor growth is delayed as well as cell proliferation, migration, and invasion (Sureban et al., 2008; Gao et al., 2015; Smith, 2015; Kharas and Lengner, 2017). In addition, murine models upregulated for *MSI1* and *K-RasG12D* are highly resistant to oxaliplatin and 5-fluorouracil (Todaro et al., 2014).

### miRNA Control

miR-137 is a tumor suppressor, which negatively regulates MSI1 and Notch/WNT signaling pathway (Smith, 2015). There is an inverse correlation between miR-137 and MSI1 expression; thus, the overexpression of miR-137 decreases *MSl1* expression reducing cell growth, colony formation, and tumor sphere growth (Liang et al., 2013; Smith et al., 2015).

### Clinical Relevance

*MSI1/2* are highly expressed in colon primary tumors and metastatic lesions in the lymph nodes; this correlates to an increased metastatic risk and poorer survival (Fan et al., 2010; Li et al., 2011). Therefore, it has been suggested that inhibition of both MSIs' RNA binding activity could fully abrogate tumor growth in CRC (Potten et al., 2003; Cheng et al., 2015; Gao et al., 2015; Lan et al., 2015; Kharas and Lengner, 2017). Besides, MSI increases colorectal cancer stem cells (CSCs) survival, migration, and resistance to 5-FU, the chemotherapy drug that constitutes the backbone of the most currently used one in colon cancer treatment (Yuqi et al., 2008; Voutsadakis, 2018; López-Cortés et al., 2019).

## ELAVL1

### General Features

Embryonic Lethal, Abnormal Vision Drosophila-Like 1 (*ELAVL1*) or Hu Antigen R (*HuR*) was the first factor to be identified for its ability to cooperate and compete with miRNA activity (Franceschini et al., 2012; Ciafrè and Galardi, 2013; Iadevaia and Gerber, 2015). ELAVL1 consists of 326 amino acids harboring three RRMs, which bind to specific mRNAs in their AU- or U-rich elements (AREs) in their 3′UTRs (López de Silanes et al., 2004b). *ELAVL1* expression changes were found to occur early during tumorigenesis (Fan and Steitz, 1998), upregulating key survival or growth-related genes by increasing both their mRNA stability and/or their protein translation (Fan and Steitz, 1998; López de Silanes et al., 2004b; Franceschini et al., 2012). For example, ELAVL1 promotes the stability and translation of COX-2 mRNA by binding to its ARE sequences located within the 3′UTR in an advanced tumor stage of CRC tissues (Fan and Steitz, 1998; Dixon et al., 2001; Denkert et al., 2006; Badawi et al., 2017). COX-2 is a major facilitator of several cellular activities (e.g., proliferation, cell death resistance, angiogenesis, and metastasis) (Fan and Steitz, 1998; Denkert et al., 2006; Jang et al., 2017; López-Cortés et al., 2019). ELAVL1 is normally found in the nucleus, where it participates in splicing and polyadenylation, but in CRC cells ELAVL1 is localized in the cytoplasm promoting mRNA stabilization of its targets (Fan and Steitz, 1998; Brennan and Steitz, 2001; López de Silanes et al., 2011; Akaike et al., 2014; Liu et al., 2018). ELAVL1 regulates numerous mRNAs that encode proteins related to proliferation, cell cycle (cyclins A2, B1, D1, p21, and p27), tumor suppressors (p53 and Von Hippel-Lindau tumor suppressor), proto-oncogene products (c-Fos and c-Myc), growth factors (IGF-1R VEGF, EGF, TGF, GM-CSF), inhibitors (p21 and p27) and signaling molecules, which are crucial (β-catenin, cyclin D1, and c-Myc) for the CRC WNT-activated pathway (Lin et al., 2017).

### Xenograft Studies

ELAVL1 enhances pathogenic gene expression necessary for cancer development (Blanco et al., 2016). This was corroborated when *ELAVL1* overexpression increased colon cancer cells growth in a nude mouse xenograft model (López de Silanes et al., 2004b; Liu et al., 2018). Subcutaneous injection of ELAVL1-overexpressing RKO cells into nude mice produced significantly larger tumors; conversely, RKO cells expressing low ELAVL1 levels significantly reduced tumor growth (López de Silanes et al., 2003). *ELAVL1* deletion in adult normal mice was lethal and several critical defects were observed: defective intestinal stem cell dynamics, villus atrophy, and defects in hematopoietic progenitor cell production (Ghosh et al., 2009). Mice lacking *ELAVL1* in myeloid-lineage cells, which include many of the innate immune system cells, showed a rapid progression of chemical-induced colitis and increased susceptibility to endotoxemia and colitis-associated cancer (Yiakouvaki et al., 2012).

### miRNAs Control

ELAVL1 levels are downregulated by miR-519, a tumor-suppressive miRNA. miR-519 promotes anti-proliferative properties in CRC cell lines by targeting and reducing ELAVL1 transcripts. This, in turn, decreases the expression of several ELAVL1 target mRNAs and markedly reduces cell proliferation (Abdelmohsen et al., 2008). ELAVL1 levels are also downregulated by miR-22, which has a more profound tumor-suppressive effect. Expression of miR-22 is inversely correlated with ELAVL1 in both CRC tissues and CRC cell lines. miR-22 directly binds to the 3′UTR of ELAVL1 leading to its inhibition, which, in turn, represses CRC proliferation and migration *in vitro* and decelerates CRC xenografted tumor growth *in vivo* (5). Conversely, Al-Haidari et al. have found that when miR-155-5p expression was reduced in serum-starved CRC cells, it decreased the expression of ELAVL1 (Al-Haidari et al., 2018).

### Clinical Relevance

Increased expression and cytoplasmic abundance of ELAVL1 is correlated with malignancy in colon cancer tissues (López de Silanes et al., 2004a; Denkert et al., 2006). Numerous studies have indicated that cytoplasmic accumulation of ELAVL1 has a link to multidrug resistance (MDR) acquired after chemotherapy and therefore causing poor prognosis in various cancer types. Accordingly, suppression of ELAVL1's cytoplasmic accumulation could increase chemotherapeutic agent accumulation and induced apoptosis, leading to increased cytotoxic effect and reversing drug resistance (Blanco et al., 2016; Lin et al., 2017). *ELAVL1* inhibition could, therefore, improve the efficacy of current therapy regimes (Badawi et al., 2017; Lin et al., 2017).

## QKI

### General Features

Quaking (QKI) is a human RBP placed on chromosome 6q26. QKI is part of STAR (signal transduction and activation of RNA) protein family and presents two specific regions (QUA1 and QUA2) and a KH domain. So far, four mRNA splice variants have been recognized: QKI-5, QKI-6, QKI-7, and QKI-7b (Kondo et al., 1999; Yang et al., 2010). The 3 well-studied isoforms (QKI5, 6, and 7) appear to have different roles in development. QKI isoforms are constructed with the same 311 amino acid body (share exons 1–6), however, their C-terminal differs from the rest (35 amino acids) (Yang et al., 2010). From all human isoforms, QKI-5 is the most abundant in colon tissues where its maximum expression is seen in the nucleus, while QKI-7 is mainly a cytoplasmic protein. QKI-6 can be found in both nuclear and cytoplasmic compartments (Yang et al., 2010; Ji et al., 2013). The cellular localization of QKI7b remains unknown due to the lack of specific antibodies (Liu Q. et al., 2013).

QKI affects several RNA-related processes (pre-mRNA splicing, mRNA stabilization and turnover, nuclear retention, miRNA processing, and circular RNA biogenesis), regulating cell cycle and differentiation, programmed cell death, development, new blood vessels formation, and cell fate determination (Nilsen and Graveley, 2010; Ji et al., 2013). To date, altered expression of the STAR proteins has been seen in several developmental defects and diseases. Concerning to CRC, Yang et al. discovered that QKI5 and QKI6 are very little expressed or even absent, acting as tumor suppressor proteins. This was associated with unusual regulation of β-catenin and p27Kip1 signaling (Yang et al., 2010). This reduction in *QKI* expression has been for an anomalous dropping of the histone variant macroH2A1.1 (Ji et al., 2013). QKI is a critical regulator of colon epithelial differentiation, whose aberrant reduction (hypermethylation) might contribute to gastrointestinal cancer initiation and facilitate colon carcinogenesis (Yang et al., 2010; Iwata et al., 2017).

### Xenograft Studies

*QKI* null mice phenotype presented several abnormalities in the vascular remodeling or vitelline vessels that make them impossible to survive later of the day 10.5. *QKI* conditional knockout mice died by the third week after birth, displaying severe hypomyelination in the central nervous system (Darbelli et al., 2016). The lethal phenotype in *QKI* knockout mice highlights the importance of this gene in the regulation of normal cellular functions (Yang et al., 2010).

### miRNAs Control

miR-574-5p negatively controls the expression of QKI6/7/7b through binding to QKI's 3'UTRs. This negatively regulation has been seen in mice and humans colorectal tissues where β-catenin and p27Kip1 signaling is affected once miR-574-5p is significant upregulated (Ji et al., 2013). Another regulator is miR-155 which downregulates *QKI* and thus promotes proliferation and invasion of CRC cells (He et al., 2015).

### Clinical Relevance

Low *QKI* expression is a risk factor for tumor recurrence after surgery. Thus, patients with low *QKI* expression had significantly poorer prognosis. Furthermore, the relapse-free survival (RFS) and overall survival of patients with stage I, II, and III CRC with low *QKI* expression was significantly shorter than those with high QKI expression. QKI could be therefore a useful clinical biomarker for predicting recurrence and prognosis (Iwata et al., 2017). In addition, if methylation-related mechanisms contribute to the inactivation of *QKI*, demethylation could be an appropriate therapeutic strategy (Yang et al., 2010; Iwata et al., 2017).

## RBM3

### General Features

RNA-binding motif protein 3 (RBM3) has been identified as a cold-shock protein. RBM is activated in cellular distress (e.g., hypothermia, hypoxia, and oxidative stress), but it is necessary for cell proliferation (Melling et al., 2016; Siesing et al., 2017). RBM3 is part of the glycine-rich RNA-binding protein family and has one RRM domain. Currently, two isoforms have been identified where the longest comprehends 157 amino acids with a molecular mass of 17 kD (Derry et al., 1995; Melling et al., 2016; Jang et al., 2017; Rappaport et al., 2017). Cold-shock proteins have been suggested to be important mediators of the caspase-independent mitotic death (CIMD) (Jang et al., 2017). RBM3 interferes the access of mRNA initiation factors to the 60S ribosome which modulates the potential activity of kinases in tumors (Chappell and Mauro, 2003; Dresios et al., 2005).

RBM3 plays a key role in carcinogenesis and proto-oncogene function. RBM3 augments mRNA stability and translation of rapidly degraded transcripts by binding to their AREs; for instance, RBM3 stabilizes COX-2, IL-8, and VEGF (Sureban et al., 2008; Venugopal et al., 2016). These cells also exhibit augmented stem cell markers via an increase in β-catenin activity. Therefore, the β-catenin signaling pathway may be regulated through alterations in the expression of *RBM3*. Interestingly, RBM3 is also regulated by hypoxia in a HIF1α independent mechanism; this provides a novel target to further examine RBM3-mediated hypoxia induced stem cell signaling (Venugopal et al., 2016).

### Xenograft Studies

*RBM3* overexpression enhanced the development of multicellular tumor spheroids in NIH3T3 mouse fibroblasts. This suggests that *RBM3* could malignantly transform cells by inducing anchorage-independent growth. However, in xenografts models *RBM3* downregulation reduces tumor growth and angiogenesis. Given the reduction in the expression of *IL-8* and the proangiogenic factors *COX-2* and *VEGF* (Sureban et al., 2008).

### miRNAs Control

It has been reported that RBM3 alters miRNA levels which in turn will modify global protein expression and thus tumor progression (Jang et al., 2017).

### Clinical Relevance

*RBM3* overexpression in HCT116 and DLD1 colon cancer cells increases proliferation and engenders hypoxia, serum deprivation and resistance to classical chemotherapeutic agents (e.g., cisplatin, doxorubicin, and paclitaxel) (Venugopal et al., 2016). It has been suggested that RBM3 is capable of increasing chemoresistance by inducing cells with high xenobiotic efflux capacity and through the induction of ATP-binding cassette (ABC) transporters (Venugopal et al., 2016). In contrast, RBM3 downregulation decreases HCT116 colon adenocarcinoma cell proliferation (Sureban et al., 2008). There is an association between RBM3 and more favorable clinic pathological parameters, the higher *RBM3* expression, the higher the disease-free survival (DFS) rate is, particularly in patients who received first line oxaliplatin-based chemotherapy (Jones et al., 2014; Venugopal et al., 2016; Jang et al., 2017; Liu Y. et al., 2017; Siesing et al., 2017; Ye et al., 2017).

Besides, a positive relationship between microsatellite instability with high expression of *RBM3* was observed (Venugopal et al., 2016; Jang et al., 2017). High microsatellite instability is commonly associated with good prognosis and right-sided colon cancer development. Melling and colleagues found that the higher the expression of *RBM3* the higher the overall survival is in CRC (stages I-III). This may be clinically relevant for the selection of patients with a likely adverse clinical course for adjuvant chemotherapy. Although, no difference in survival was seen for rectal carcinomas (Melling et al., 2016). Noteworthy, Wang and colleagues found that *RBM3* positive expression correlates with an improved prognosis in young CRC patients (Wang M. J. et al., 2015).

## CELF1

### General Features

CUGBP Elav-like family member 1 (CELF1), is a multifunctional RBP that generally binds mRNAs through GU-rich elements in the 3′-UTRs or coding regions of its targets. CELF1 forms part of a family named CELF (CELF1, CELF2, CELF3, CELF4, CELF5, and CELF6). All the family members possess a divergent domain loaded with alanine and glutamine residues and three RRMs, two near the N-terminal region and one located at the C-terminal domain. CELF1 promotes and represses RNA splicing and mRNA translation (Kim and Gorospe, 2008; Vlasova et al., 2008; Vlasova-St. Louis and Bohjanen, 2011; Yang et al., 2014; Liu et al., 2015). The three of them recognize different motifs and arrangements, which gives specificity and a wide range of binding partners. CELF1 regulates protein expression implicated in the tight junction (TJ) and gut barrier function. For instance, CELF1 represses occludin translation by increasing occludin mRNA recruitment to processing bodies, resulting in dysfunction of the epithelial barrier. Interestingly, CELF1 and ELAVL1 compete for the same occludin 3′UTR binding element, competitively regulating occludin translation and in opposite directions (Yang et al., 2014; Liu et al., 2015). CELF1 also regulates intestinal epithelial homeostasis by modulating intestinal epithelial cells (IECs) proliferation, apoptosis and cell-to-cell interaction (Cui et al., 2012; Liu et al., 2015). Increased levels of cellular *CELF1* desensitize IECs to apoptosis, whereas *CELF1* silencing increases the sensitivity of IECs to apoptosis (Cui et al., 2012; Tu et al., 2015).

### Xenograft Studies

In a mouse fasting model, a reduction in the proliferating crypt cell population and a decrease in the lengths of villi and crypts were correlated with a significant increase in the levels of *CELF1*. This suggests the involvement of *CELF1* in the pathogenesis of intestinal mucosal atrophy (Madison et al., 2013, 2015; Liu et al., 2015).

### miRNA Control

CELF1 is repressed by the tumor suppressor miR-503 in IECs. CELF1 abundance is regulated by miRNA-503 mostly by binding to sites located in CELF1 coding region (Ciafrè and Galardi, 2013; Yang et al., 2014; Liu et al., 2015).

## CELF2

### General Features

CUGBP Elav-like family member 2 (CELF2) is a ubiquitously expressed protein of 490 amino acids located on chromosome 10p13–p14 (Choi et al., 1999; Lichtner et al., 2002; Ramalingam et al., 2012). CELF2 regulates several RNAs at different posttranscriptional levels: alternative splicing (e.g., Tau and troponin T), RNA editing (e.g., apolipoprotein B), RNA stability, and mRNA translation (e.g., cyclooxygenase-2 and Mcl1) (Ramalingam et al., 2012). CELF2 is expressed in the nucleus of intestinal epithelial cells acting as a tumor suppressor protein (Natarajan et al., 2008). CELF2 has at least three identified isoforms, each of them with differential expression levels in human colon cancer cells (Ramalingam et al., 2008). *CELF2* overexpression results in reduced colony formation in CRC cells. CELF2 attaches to AREs of COX-2 3′UTR increasing COX-2 mRNA stability but inhibiting its translation. Reduction of COX-2, in turn, decreases PGE2 known to modulate cell proliferation and tumor invasion in many cancer types (Sureban et al., 2008; Ramalingam et al., 2012). *COX-2* is upregulated in colorectal adenomas, thereby suggesting that CELF2 might prevent cancer development by inhibiting COX-2 and PGE2. These data suggest that *CELF2* expression may be deleterious to cancer cells (Ramalingam et al., 2012).

## IGF2BP1-3

### General Features

The mammalian IGF2 mRNA-binding protein family (IGF2BP) comprises three RNA-binding proteins (IGF2BP1-3) with a conserved domain structure including four K homology (KH) domains and two RRMs (Ross et al., 2001; Dimitriadis et al., 2007; Lederer et al., 2014). IGF2BPs exhibit different expression patterns despite their high degree of likeness and show distinct RNA-binding properties and are associated with variable target transcripts. IGF2BP1 stabilizes the MYC mRNA by shielding it from ribonuclease cleavage when binding to the coding region instability determinant (Lederer et al., 2014). Thereby, it prolongs the half-life of MYC mRNA up to 8 fold, promoting tumor cell proliferation and survival (Ross et al., 2001; Dimitriadis et al., 2007; Hamilton et al., 2013; Lederer et al., 2014). IGF2BP1 also regulates CD44, ALCAM, AMIGO2, MCAM, CD24, dysadherin, and MMP1 mRNAs that encode proteins of cell adhesion and invasiveness (Dimitriadis et al., 2007; Vainer et al., 2008). In addition, IGF2BP1 binds to and stabilizes F-box protein βTrCP1 whose continued activation in CRC is well established by suppressing apoptosis via NF-κB activation (Dimitriadis et al., 2007; Hamilton et al., 2013).

Concerning IGF2BP2, it has been shown that this RBP controls NRAS, PINCH2, and MURF-3 expression which are responsible for carcinogenesis and cellular mobility (Lederer et al., 2014; Ye et al., 2016). In addition, IGF2BP2 targets RAF1 mRNA which is an essential component of MAPK pathway activation upon (MEK)1/2 phosphorylation. MEK1/2, in turn, phosphorylate and activate extracellular-related kinase (ERK)1/2. ERK1/2 regulate downstream pathways involved in survival and cell proliferation (Ye et al., 2016).

Regarding IGF2BP3, this protein contributes to RNA trafficking and stabilization, cell development and division, migration and adhesion (Lochhead et al., 2012; Lin et al., 2013; Lederer et al., 2014; Kumara et al., 2015). Also, *in vitro* studies have shown that IGF2BP3 promotes tumor cell survival, proliferation, anchorage-independent growth, chemoresistance migration and invasiveness (Lederer et al., 2014). It has been demonstrated that IGF2BP3 along with HNRNPM controls the fate of cyclin D1, D3, and G1 encoding transcripts in the nucleus (Li et al., 2009; Lederer et al., 2014). Cyclins are key components of the cell cycle and disorders of their function can lead to carcinogenesis. IGF2BP3 also regulates the gene expression of IGF-II, which binds to and activates IGF-I. Thus, IGF-I induces a cell to begin cell division in an alter manner which in turn causes excessive cell proliferation and cancer (Lin et al., 2013).

### Xenograft Studies

IGF2BP1 plays an essential role for normal intestinal morphogenesis since deficient mice exhibit dwarfism and severe histological abnormalities in small (villous hypoplasia) and large intestine (short and irregular crypts in the colon) (Dimitriadis et al., 2007). On the contrary, *IGF2BP1* overexpression promotes tumor-cell growth in CRC (Hamilton et al., 2013). *IGF2BP2* knock-out mice possess a higher frequency of autoantibody response to IGF2BP2/p62 in colon cancer, although the mechanisms and its role in CRC carcinogenesis are still unknown (Ye et al., 2016). Ectopic expression of *IGF2BP3* enhances tumor cell aggressiveness in transgenic animals (Dimitriadis et al., 2007; Lederer et al., 2014).

### miRNAs Control

No miRNA has been reported to inhibit any member of the IGF2BP family. However, these RBPs protect some mRNAs from miRNA attack. For instance, IGF2BP1 protects Beta-transducin repeats-containing protein 1 (βTrCP1) mRNA, an important player in signal transduction, from miR-183-directed turnover (Elcheva et al., 2009; Ciafrè and Galardi, 2013). Also, IGF2BP2 regulates RAF1 (proto-oncogene) expression by blocking its degradation by miR-195 (Ye et al., 2016).

### Clinical Relevance

The suppression of apoptosis via NF-κB activation originated by IGF2BP1 in tumors are linked to unfavorable clinical outcomes in CRC patients. These patients present propensity toward early dissemination, poor response to therapy and increased tumor aggressiveness. On the contrary, the absence of IGF2BP1 expression is an independent favorable prognostic factor for survival (Dimitriadis et al., 2007; Vainer et al., 2008; Hamilton et al., 2013). CRC patients also present a high antibody response to IGF2BP2, making this protein a possible biomarker for diagnosis and prognosis (Liu W. et al., 2013). Furthermore, IGF2BP2 may be important for chemoresistance and recurrence of the disease, given its participation in the maintenance of CSCs (Degrauwe et al., 2016; Jang et al., 2017). Concerning IGF2BP3 clinical relevance, it has been shown that this protein is a marker for aggressiveness, poor differentiation and tumor progression and it is related with an unfavorable prognostic and short survival times (Lochhead et al., 2012; Lederer et al., 2014; Chen et al., 2017). Also, IGF2BP3 positive patients have a nearly 11-fold increased risk of distant metastases (Li et al., 2009; Lin et al., 2013; Wei et al., 2015; Chen et al., 2017). This strong correlation suggests, that IGF2BP3 plays an important role in epithelial-mesenchymal transition (EMT) (Li et al., 2009; Lin et al., 2013; Wei et al., 2015; Chen et al., 2017).

## ESRP1

### General Features

Also named RBM35A, epithelial splicing regulatory protein 1 (ESRP1) contains three putative RRMs, which are mutational hotspots of primary colon tumors with microsatellite instability (MSI) causing rapid degradation of the mutated transcripts (Leontieva and Ionov, 2009; Deloria et al., 2016; Mager et al., 2017). ESRP1 controls alternative splicing and regulates mRNA stability and translation of several mRNAs (Fagoonee et al., 2017). For example, ESRP1 has been identified as a key regulator for Ig-like III domain variant splicing of the fibroblast growth factor receptor 2 (FGFR2). Also, ESRP1 regulates transcript variants from genes associated with EMT such as *CD44, ENAH*, and *CTNND1* (p120-catenin) (Deloria et al., 2016). ESRP1 is a tumor suppressor in CRC due to their ability to regulate translation of several cancer-related genes by binding to their mRNA 5′UTRs. In addition, ESRP1 suppresses cancer cell motility through distinct mechanisms during EMT (Leontieva and Ionov, 2009; Deloria et al., 2016; Fagoonee et al., 2017). Ectopic expression of ESRP1 protein resulted in suppression of tumorigenic potential of LS180 colon cancer cells (Leontieva and Ionov, 2009). *ESRP1* is negatively regulated by mesenchymal transcription factors such as SNAIL, ZEB1, and ZEB2 (Mager et al., 2017).

Despite its role as a tumor suppressor, Fagoonee et al. recently demonstrated a pro-metastatic function of ESRP1 (Fagoonee et al., 2017). ESRP1 contributes to anchorage-independent growth of CRC cells, when Caco-2 cells are grown in suspension, enhances FGFR1/2 signaling, supports constant *Akt* phosphorylation and *Snail* upregulation. FGFR or PI3K/Akt inhibition reverted the pro-oncogenic phenotype of ESRP1 upregulation. High *ESRP1* expression may stimulate cancer epithelial cell growth in the colon, as well as, at distant sites promoting CRC progression (Fagoonee et al., 2017).

### Xenografts Studies

ESRP1 has a key role in intestinal homeostasis and disease in mice (Mager et al., 2017). Partial loss of *ESRP1* function impairs intestinal epithelial barrier integrity, increases susceptibility to colitis and alters CRC development. In addition, *ESRP1* overexpression has been correlated with liver macrometastasis in murine models, probably due to its ability to promote cancer cell growth at distant sites (Fagoonee et al., 2017).

### Clinical Relevance

*ESRP1* expression is associated with a favorable overall survival outcome in CRC patients. On the contrary, loss of *ESRP1* expression negatively correlates with CRC patient survival (Mager et al., 2017). Decreased *ESRP1* expression might also indicate the presence of EMT and thus disease progression and metastasis (Deloria et al., 2016). In addition, by upregulating Snail expression, ESRP1 has been associated with poor prognosis and shortened relapse-free survival (Fagoonee et al., 2017).

## TTP

### General Features

Tristetraprolin (TTP) also called ZFP36 or TIS11 forms part of a family of tandem Cys3His zinc finger proteins (Lai et al., 1999; Sharma et al., 2013; Sobolewski et al., 2015; Lee et al., 2018). TPP is mainly cytoplasmic, where interacts with stress granules (SGs), regulates mRNA stability and promotes degradation of inflammatory cytokines, proto-oncogenes and growth regulatory genes (Carrick and Blackshear, 2007; Cha et al., 2011; Lee et al., 2013). TTP functions as a tumor suppressor by inhibiting expression of cancer-related genes that encode AREs in their mRNA 3′UTRs. TTP target mRNAs encode inflammatory cytokines, cell growth factors, angiogenesis, apoptosis, and differentiation-related factors. For instance, TTP downregulates VEGF levels by reducing VEGF mRNA accumulation; this, in turn, decreases angiogenesis and reduces CRC growth (López de Silanes et al., 2003; Lee et al., 2010, 2018). TTP also regulates the expression of cancer-related proteins (Fos, Myc, COX-2, cIAP2, E2F1, Bcl-2, Mcl-1, LATS2, Lin28, and Cyclin D1), which contribute to inflammation, apoptosis, and angiogenesis in CRC (Lee et al., 2010, 2018; Sobolewski et al., 2015). Accordingly, *TTP* downregulation occurs at early stages of tumorigenesis and ectopic expression of *TTP* in CRC attenuates cell proliferation (Sobolewski et al., 2015).

### Xenografts Studies

The inverse correlation between the expression levels of *TTP* and *VEGF* has been seen in nude mice, where tumor growth and angiogenesis are inhibited by TTP-mediated VEGF downregulation (Lee et al., 2010). Besides, *TTP* knockout mouse model develops multiple inflammatory syndromes due to the increased expression of tumor necrosis factor, *COX-2* and other pro-inflammatory proteins (Sobolewski et al., 2015).

### miRNA Control

miR-29a downregulates TTP in a breast cancer model and is known to be upregulated in colon cancer (Sobolewski et al., 2015).

### Clinical Relevance

Fallahi et al. have shown that *TTP* reduction is associated with poor prognosis, tumor aggressiveness and necrosis (Fallahi et al., 2014). The pharmacologic activation of *TTP* may limit colon cancer growth when patients present resistance to anti-VEGF therapies (Lee et al., 2010). In this regard, some therapies have been developed to activate *TTP*. For instance, Resveratrol, a natural anti-cancer compound, induces cellular apoptosis and decreases migration and invasion by activating *TTP* and regulating other cancer pathways (MYC, KRAS, and FOS) (Lee et al., 2018). Another agent aiming to restore *TTP* expression in cancer cells is Vorinostat® (SAHA), already in phase 1 clinical trial (Sobolewski et al., 2015). Another option to increase *TTP* expression is the use of histone deacetylase inhibitors (HDAC inhibitors), which can restore *TTP* expression at the transcriptional level (Sobolewski et al., 2015).

## hnRNPs

### General Features

Heterogeneous nuclear ribonucleoproteins (hnRNPs) are normally localized in the nucleus; however, some may shuttle between the nucleus and cytoplasm due to their nuclear export signals. They are known as pre-mRNA/mRNA binding proteins that participate in important cellular mechanisms, such as DNA repair, response to hypoxia, splicing, nucleocytoplasmic transport, apoptosis and transcriptional and translational regulation (Ushigome et al., 2005; Hope and Murray, 2011; Lai et al., 2016). Quantitative and qualitative alterations of hnRNPs have shown to disturb cellular functions and facilitate malignant transformation (Ushigome et al., 2005).

To date, at least 20 major hnRNP proteins, from hnRNP Al to U, have been identified in human cells (Ushigome et al., 2005). All members of the hnRNP family share a similar protein structure, consisting of at least one RRM combined with other auxiliary domain: RGG box or the acidic domain responsible for protein-protein interactions (Lai et al., 2016). Given that all hnRNPs belong to the same family, their phenotypic impact is likely similar (Hope and Murray, 2011; Budak et al., 2017). Despite their great importance, few studies have been focused on cancer and much less on CRC. Table 2 summarizes their function and effect in CRC.

**Table 2:** Function and effect of hnRNPs in CRC.

**hnRNP**	**Function**	**Effect in CRC**	**Reference**
Al	- Unwinds intramolecular folded-back quadruplex structures of telomere repeats and G-rich short tandem repeats (STRs). - Abrogates DNA synthesis arrest. - Promotes a protective effect against apoptosis.	- A potential biomarker. It has a significant cytoplasmic immunoreaction in tumor cells.	Ushigome et al., 2005; Zhang et al., 2006; Hope and Murray, 2011
A18	- Promotes inflammatory responses when present extracellularly.	- Higher hnRNPA18 expression in CRC cells could be used as an independent prognostic marker.	Sakurai et al., 2014; Chang et al., 2016; Jang et al., 2017
D	- Destabilizes RNA and regulates expression of pro-inflammatory Cytokines, proto-oncogenes, and regulators of apoptosis, and the cell cycle. - Enhances mRNA stability and translation.	- Indirectly regulates cancer-related mRNAs by inhibiting Dicer-mediated mature miRNA formation. HnRNPD binds to Dicer mRNA reducing its stability. An inverse correlation between Dicer and hnRNPD expression has been observed in CRC tissues.	Dixon, 2004; Zucconi and Wilson, 2011; Ciafrè and Galardi, 2013; Dai et al., 2019
DL	- Acts as a transcriptional regulator. - Promotes transcription repression. - Stimulates transcription activation in differentiated myotubes.	- Confers growth advantage through its ability to promote cell cycle progression.	Balasubramani et al., 2006; Rappaport et al., 2017
F	- Plays a role in the regulation of alternative splicing events. - Binds G-rich sequences in pre-mRNAs and keeps target RNA in an unfolded state.	- Involved in early CRC genesis.	Balasubramani et al., 2006; Rappaport et al., 2017
H	- Mediates pre-mRNA alternative splicing regulation.	- hnRNPH is associated with good prognosis, especially in left-sided (distal) colonic tumors and rectal tumors.	Hope and Murray, 2011; Rappaport et al., 2017
I	- Activates exon skipping of its own pre-mRNA during muscle cell differentiation.	- Silences Notch signaling pathway, which is a critical mediator of stem cell proliferation and differentiation of colonic epithelium.	Hope and Murray, 2011; Jin et al., 2017; Rappaport et al., 2017
K	- Plays an important role in TP53 response to DNA damage, acting at both transcription activation, and repression.	- Could be used as a poor prognosis marker. Altered expression and cellular localization correlates with CRC tumor stage.	Hope and Murray, 2011; Guo et al., 2012; Sugimasa et al., 2015; Zhang et al., 2016; Budak et al., 2017; Rappaport et al., 2017
M	- Acts as a receptor for carcinoembryonic antigen in Kupffer cells - Initiates a series of signaling events leading to tyrosine phosphorylation of proteins and induction of IL-1 alpha, IL-6, IL-10, and tumor necrosis factor alpha.	- Positively correlates with proliferation, invasion and metastasis of CRC cells.	Chen et al., 2013
L	- Involved in the synthesis of new blood vessels.	- Promotes angiogenesis in CRC cells.	Hope and Murray, 2011
Q	- Promotes MYC mRNA stability - Modulates the posttranscriptional C to U RNA-editing of the APOB mRNA.	- Increases cell proliferation and contribute to tumorigenesis.	Lai et al., 2016; Rappaport et al., 2017
U	- Repairs double-strand DNA.	- Aberrantly found in the nucleus of CRC cells, compared with normal colonic epithelium.	Hope and Murray, 2011

## TIA1

### General Features

T-cell intracellular Antigen-1 (TIA1) is a cytoplasmic granule-associated RBP which contains three RRMs (Zlobec et al., 2010; Hamdollah Zadeh et al., 2014; Yang et al., 2014). TIA1 is linked to multiple biological processes associated with RNA metabolism and plays an important role in the regulation of gene expression, predominantly under conditions of cellular stress (Liu et al., 2014; Yang et al., 2014). TIA1 is alternatively spliced in exon 5 to form two isoforms (short and long), both of them reported to be expressed in cytolytic cells. TIA1 inhibits both transcriptional and posttranscriptional events of many transcripts involved in cancer cell proliferation, apoptosis, angiogenesis, invasiveness, and metastasis as well as in immune evasion (Hamdollah Zadeh et al., 2014; Liu Z. P. et al., 2017). For example, TIA1 can promote cell apoptosis by regulating Fas alternative splicing, while also enhancing NK cell cytotoxic activity (Zlobec et al., 2010; Liu Z. P. et al., 2017). TIA1 is downregulated at the protein level in CRC, which is therefore considered as a tumor suppressor RBP (Liu Y. et al., 2017).

### Xenograft Studies and miRNA Control

TIA1 is a direct target of miR-19a in CRC, where is highly expressed (Liu Y. et al., 2017). miR-19a is part of a family known as mir17-92, which possess several cellular functions as survival, proliferation, differentiation, and formation of new blood vessels (Olive et al., 2009). This miRNA promotes cell proliferation and migration *in vitro* and accelerates tumor growth in xenografed mice. miR-19a binds directly to the 3′-UTR of TIA1 mRNA inhibiting TIA1 cancer suppressive features. Suppression of miR-19a activity could increase cellular levels of TIA1, therefore impairing cancer-related cellular processes (Liu Y. et al., 2017).

### Clinical Relevance

TIA1 is a robust prognostic immunological biomarker in CRC and particularly in tumors with marked cytotoxic CD8+ tumor-infiltrating lymphocytes (TILs). Increased numbers of TIA1 positive TILs is associated with an improved clinical outcome representing an independent prognostic factor (Zlobec et al., 2010). TIA1 can also be used to supplement prognostic information related to TNM (tumor, node, and metastases) stage and adjuvant therapy in mismatch repair-proficient colorectal cancer patients (Liu Y. et al., 2017).

## KHDRBS1

### General Features

KH RNA binding domain containing signal transduction associated 1 (KHDRBS1) protein or Src-associated in mitosis 68 kDa protein is part of STAR family KH domain-containing RBPs (Sánchez-Jiménez and Sánchez-Margalet, 2013). It is a substrate for Src kinases, which are often activated in human cancers. KHDRBS1 is usually a nuclear protein, which is a mitogene and is also involved in transformation and tumorigenesis. This RBP plays a major protagonist in the life cycle of RNA molecules. Besides, KHDRBS1 regulates the alternative splicing of several genes, most of them involved in human cancer, such as *CD44, Bcl-xl, Sgce, SMN2, SF2/ASF*, and *Cyclin D1*. KHDRBS1 also participates in early cellular responses to DNA damage by controlling the signaling cascade that links DNA damage recognition in the nucleus to NF-κB liberation and activation in the cytoplasm. Accordingly, *KHDRBS1* downregulation promotes self-destruction of colon cancer cells under exposure to DNA-damaging agents. KHDRBS1 is therefore important for CRC development and survival (Fu et al., 2016).

### Xenograft Studies

*KHDRBS1* null mice produced delays in colon tumor growth, metastasis, cell migration, and extremely sensitiveness to agents that cause DNA damage (Lukong and Richard, 2007; Fu et al., 2016).

### Clinical Relevance

Several studies have reported *KHDRBS1* to be overexpressed in CRC tissues. KHDRBS1 nuclear localization and overexpression is correlated with poorly differentiated cancer cells, advanced T, N, and M1 stage. Poor prognosis and a higher risk of recurrence have been seen in patients with high levels of this protein or nuclear localization (Liao et al., 2013; Fu et al., 2016).

## CPEB4

### General Features

Cytoplasmic Polyadenylation Element Binding Protein 4 (CPEB4) is a ubiquitous cytoplasmic zinc-finger RBP (Zhong et al., 2015; He et al., 2017; Rappaport et al., 2017). *CPB4* gene is localized on chromosome 5q35 and encodes a protein composed of 729 amino acids (He et al., 2017). CPEB4 can modulate the cellular epigenetic profile and influence several biological activities such as cell proliferation and differentiation, chromatin-remodeling, and chromosome segregation (Zhong et al., 2015; He et al., 2017). Furthermore, CPEB4 recruits translational repression or polyadenylation machinery, which targets mRNAs that regulate mitotic and meiotic cell cycle and senescence (Cortés-Guiral et al., 2017; He et al., 2017). Importantly, *CPEB4* is highly expressed in a variety of malignant tumors, including CRC, promoting tumor proliferation, invasion, migration, and vascularization (Zhong et al., 2015; Cortés-Guiral et al., 2017; He et al., 2017). CPEB4 can also influence apoptosis of tumoral cells by modulating the expressions of B-cell lymphoma extra-large (Bcl-XL) and B-cell lymphoma-2-associated X (Bax) proteins. *CPEB4* knockdown increases Bax expression but decreases Bcl-XL expression. Changes in the homeostatic balance of Bax and Bcl-XL lead to a deregulation of apoptosis during tumor development. In addition, three parameters are considered as prognostic markers in CRC: (i) age, (ii) body tumor location, and (iii) Bax/Bcl-2 ratio (Zhong et al., 2015).

### miRNA Control

Recent studies have shown miR-203, a tumor suppressive miRNA, significantly decreased in colorectal cancers. miR-203 inhibits cancer growth and enhances cell apoptosis by suppressing CPEB4 expression post-transcriptionally (Zhong et al., 2015; Cortés-Guiral et al., 2017). Hence, miR-203-mediated CPEB4 degradation might be a novel strategy in CRC treatment (Zhong et al., 2015).

### Clinical Relevance

*CPEB4* is aberrantly expressed in CRC tissues and correlates with tumor progression and poor overall survival in CRC patients. Thus, detecting CPEB4 expression in CRC tissues or peripheral blood might be used as an additional parameter to identify patients with a high risk of tumor invasiveness and/or metastasis. Patients with these characteristics could be considered for more personalized and aggressive treatment (Zhong et al., 2015; He et al., 2017).

## AGO

### General Features

The Argonaute (AGO) proteins are fundamental components of RNA-induced silencing complexes (RISC) and RNA interference (RNAi) machinery, which induce endonuclease cleavage of mRNA and miRNA passenger strand. Currently, two subfamilies with 4 Argonaute-like proteins within each have been described: (i) eIF2C/AGO subfamily with AGO1, AGO2, AGO3, and AGO4 and (ii) PIWI subfamily with PIWIL1, PIWIL2, PIWIL3, and PIWIL4. AGO subfamily genes are ubiquitously expressed and are regulated in a cell-context-dependent manner (Li et al., 2010; Rüdel et al., 2011).

Small interfering RNAs (siRNAs) and/or miRNAs are used by Ago proteins as silencing mechanisms in both transcriptional and posttranscriptional processes. Overexpression of AGO members has been associated with excessive growth and programmed cell death inhibition of cancer stem cells. Specifically, increased expression of *AGO2-4* and *PIWIL4* has been associated with colon cancer occurrence in advanced tumors with distant metastasis (Li et al., 2010).

## CSDE1

### General Features

Cold shock domain containing E1 (*CSDE1*), formerly named Upstream of the *NRAS* (UNR), is an RBP composed of 798 amino acids with 5 cold shock domains. *CSDE1* gene is located on chromosome 1p13.2 upstream of the *NRAS* locus. CSDE1 has been shown to regulate mRNA stability and translation of several oncogenes, such as *c-MYC, c-FOS, VIM, PTEN*, among many others, in melanoma, breast, pancreatic and prostate cancer (Grosset et al., 2000; Evans et al., 2003; Chang et al., 2004; Wurth et al., 2016). Recently, Martinez-Useros et al. have reported key oncogenic features of CSDE1 in CRC. According to this study, *CSDE1* is overexpressed in several CRC-derived cell lines, paired tumor samples, colonospheres and cell cultures originated from metastatic lesions. In contrast, *CSDE1* downregulation increases sensitivity to apoptosis and decrease invasiveness, cell viability and migration by an EMT regulation process. In addition, *CSDE1* expression positively correlates with *c-MYC* expression in CRC samples and cell lines, supporting its role as a CRC oncogene (Martinez-Useros et al., 2019).

### Clinical Relevance

Martinez-Useros et al. also demonstrated a possible role of *CSDE1* as a clinical marker, predicting poor outcome of CRC patients. Patients with high *CSDE1* expression presented shorter mean survival than patients with low expression. Although the sample size to achieved this conclusion was small, an *in silico* analysis using The Cancer Genome Atlas (TCGA)-Colorectal Cancer Dataset showed similar results: patients with high *CSDE1* expression presented shorter mean progression-free survival than patients with low *CSDE1* expression (Martinez-Useros et al., 2019).

## Common Features of all Colon Cancer Related RBPs

RBPs are pivotal members of the posttranscriptional process and key players of RNA regulons. Within this notion, RBPs regulate mRNAs that encode functionally related proteins through a RNP-driven mechanism. Genomic alterations of RBPs could therefore produce erroneous conformation of these RNP complexes leading to atypical protein expression and cancer development (Castello et al., 2016; Wurth et al., 2016; Pereira et al., 2017). To shed light on CRC RNA regulons, we studied CRC RBPs common features related to their protein domains, protein-protein interactions, and common RNA targets. In addition, we also organized CRC RBPs oncogenic capabilities into the hallmarks of cancer (Hanahan and Weinberg, 2011).

**Figure 1:**
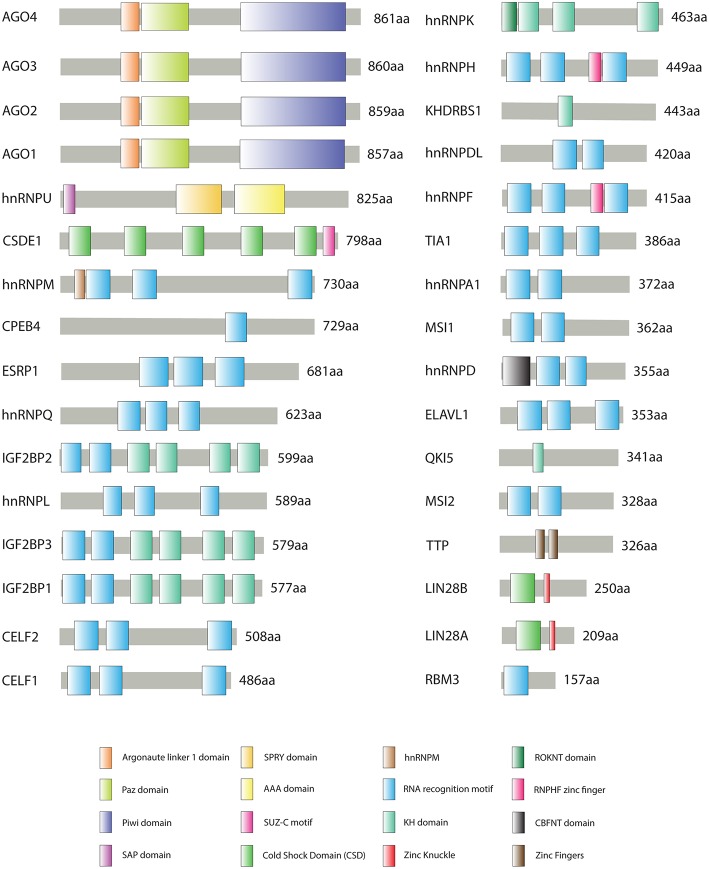
Schematic representation of colorectal cancer (CRC)-associated RNA-Binding Proteins (RBPs) structural domains according to UniProt database (https://www.uniprot.org). Sixteen structural domains represented by colored boxes, protein names and scaled lengths are shown.

**Figure 2:**
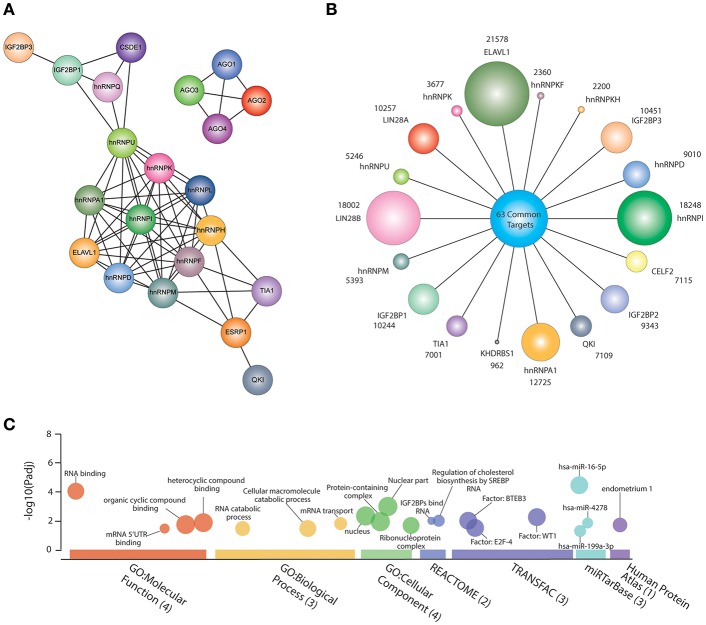
Common features of colorectal cancer (CRC)-related RNA-Binding Proteins (RBPs). **(A)** CRC RBPs RNA targets according to POSTAR2 database. Circle sizes are correlated with the number of targets of each protein; shared RNA targets are shown in the middle. **(B)** A network showing CRC RBPs protein-protein interactions from experimental data and databases (interaction score: > 0.9). **(C)** Gene set enrichment analysis showing all significantly enriched terms concerning Gene Ontology (Molecular Function, Biological Process, and Cellular Component), Reactome, Transfac, miRTasBase, and Human Protein Atlas through g:Profiler (https://biit.cs.ut.ee/gprofiler/gost). The size of the circle is correlated with the number of genes overrepresented in association with certain type of molecular function or biological process. *P*-value adjusted (Padj) for multiple testing using Benjamini-Hochberg method.

Figure 1 shows all CRC-associated RBPs and their protein domains according to UniProt database (https://www.uniprot.org/) (The UniProt Consortium, 2019); we detected 16 protein domains, of which 13 are RNA-binding domains: RRM, CSD, KH domain, Zinc finger, etc. RRM is the most prevalent domain of the CRC-related RBPs, followed by KH domain and CSD. To detect protein-protein interactions, we generated an interaction network using STRING database (Szklarczyk et al., 2017) with experiment scores > 0.9. Seventeen out Thirty-Two RBPs form a complex interaction network (Figure 2A); we also observed interactions between all AGO members.

Over the past decade, high-throughput technologies have been developed to identify RBP biding sites *in vivo* (ultraviolet crosslinking followed by immunoprecipitation and sequencing—CLIP) and mRNA translation performance (Ribosome profiling—Ribo-seq) (Ingolia et al., 2009). Recently, Zhu et al. integrated several datasets from these high-throughput technologies to investigate post-transcriptional regulatory processes mediated by RBPs through a database named POSTAR2 (http://lulab.life.tsinghua.edu.cn/postar/). This allowed us to identify all RNA targets of 18 out 32 CRC-related RBPs (Figure 2B). For example, ELAVL1 could bind to 21578 RNAs, while KHDRBS1 interacts with 962. A *post-hoc* analysis revealed 63 common targets (Supplementary Table 1). Interestingly, these RNAs encode proteins or participate in RNA-related processes, such as RNA binding or mRNA transport (Figure 2C), adding another layer to the RNA regulon model: interacting RBPs that regulate RNAs implicated in RNA-associated processes. In fact, 30% of these common targets (*n* = 19) are also RBPs (Figure 2C) (Culjkovic-Kraljacic and Borden, 2018). However, some well-known cancer driver genes, such as *MYC* and *COX-2* are common targets of 11 and 7, respectively out of 18 CRC RBPs.

**Figure 3:**
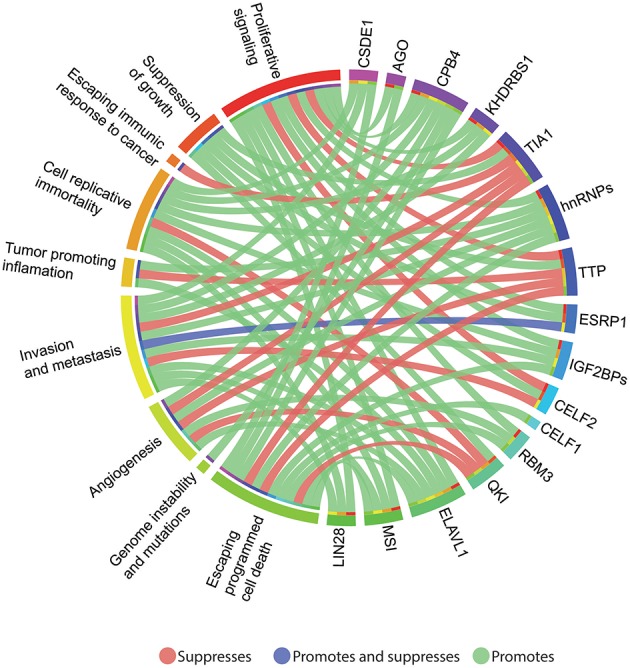
Circos plot (http://circos.ca/intro/features/) depicting the relationships between colorectal cancer (CRC) RNA-binding proteins (RBPs) and the hallmarks of cancer. Outer circle: right, CRC RBPs and left, hallmarks of cancer associated with each studied protein. Green-colored links indicate a cancer promoting activity, while red links, suppression, and the blue-colored link denotes both, promotion and suppression activity.

Finally, to better understand the oncogenic potential of the aforementioned RBPs, their capabilities were organized according to the hallmarks of cancer. As shown in Figure 3, most RBPs (11 out of 16) act as oncogenes and only 5 (TIA1, TTP, QKI, ESRP1, CELF2, and QKI) present tumor suppressive abilities. Worthy of note, TIA1 is the only RBP that suppresses the ability of cancer cells to escape immune response by enhancing NK cell cytotoxic activity (Zlobec et al., 2010; Liu Z. P. et al., 2017); also, CPEB4 is the only RBP that promotes genome instability by influencing chromatin-remodeling (Negrini et al., 2010; Zhong et al., 2015; He et al., 2017).

## Concluding Remarks and Perspectives

Several efforts have been devoted to decipher the molecular basis behind the carcinogenesis process. Most of this knowledge was achieved by studying DNA and protein function, leaving post-transcription under-investigated. In this regard, RBPs play a significant role in controlling gene expression through complex interconnected networks named RNA regulons. Consequently, RBPs alterations could greatly disrupt cellular homeostasis promoting cancer development.

We believe this work offers a comprehensive list of all CRC-associated RBPs, including their individual features to common interactions and targets. However, we only found in the literature 35 RBPs out of 1,393 having oncogenic roles in CRC; a comprehensive characterization of RBPs is therefore still missing. To shed light on this matter, not only the identification of the CRC-RBPome should be prioritized, but also its dynamics concerning CRC RNA regulons implicated in cancer progression. As we shown in Figure 2, these interactions are highly complex and more research is needed to identify key therapeutic interactions. To date, no RBP-based drug has been developed to treat CRC, according to the Open Target Platform (https://www.targetvalidation.org).

RNA-based research is generating large datasets from high-throughput technologies, such CLIP, Ribo-seq or interactome capture. To successfully understand this complexity, all this data should be compiled and analyzed by bioinformatics and systems biology approaches, such as POSTAR2 or the ones developed by the RNA Bioinformatics Center (Backofen et al., 2017). In addition, large datasets, such as The Cancer Genome Atlas (Tomczak et al., 2015), the Human Protein Atlas (Pontén et al., 2008) or Depmap (Yu et al., 2016) could be exploited to identify key RBPs that, with further research, could be used as CRC biomarkers or new therapeutic targets.

## Author Contributions

JG-C and SG conceived the subject and wrote the manuscript. CP supervised the project and provided conceptual advice and valuable scientific input. AL-C, IA-C, PG-R, AP-V, VY, AZ, and PL made a substantial contribution to the structure and design of the manuscript.

### Conflict of Interest Statement

The authors declare that the research was conducted in the absence of any commercial or financial relationships that could be construed as a potential conflict of interest.
